# Pulse Electrodeposited Ni-26 at. %Mo—A Crossover from Nanocrystalline to Amorphous

**DOI:** 10.3390/nano11030681

**Published:** 2021-03-09

**Authors:** Jiongxian Li, Yinong Shi, Xiuyan Li

**Affiliations:** 1 Shenyang National Laboratory for Materials Science, Institute of Metal Research, Chinese Academy of Sciences, Shenyang 110016, China; jiongxianli15s@imr.ac.cn (J.L.); xyli@imr.ac.cn (X.L.); 2 School of Materials Science and Engineering, University of Science and Technology of China, Hefei 230026, China

**Keywords:** composite, hardness, nanocrystalline, grain boundary activities, crystallization, amorphous

## Abstract

A Ni-26 at. %Mo alloy with a composite structure of nanocrystalline and amorphous was synthesized by pulse electrodeposition. The composite structure was composed of mixed regions of amorphous and nanograins divided by a nanocrystalline interface network, which significantly suppressed grain coarsening and shear banding that would otherwise deteriorate mechanical properties of extremely fine nanograined metal. Plastic strain induced significant crystallization accompanied by Mo diffusion from mixed regions to nanograined interfaces. As a result, the Ni-26 at. %Mo alloy exhibited a superior hardness to its nanograined counterparts. The present work demonstrates an example of enhancing mechanical performance with hybrid structures crossover from nanocrystalline to amorphous.

## 1. Introduction

Nanocrystalline (NC) and amorphous metallic materials represent two classes of novel materials that have attracted much endeavors in recent years for their exceptional performances [1,2,3,4]. Despite differences in structure and properties, similarities do exist in these two types of materials, especially when grain size of NC materials approaches the size limit [5,6,7,8]. It has been recognized that there is a crossover from grain boundary (GB) mediated activities to glass-like deformation when grain size of NC metals approaches an extremely fine region or vise-versa [5,6]. Strain localization has frequently been observed in deformation of NC materials with decreasing grain size [9,10]. Either GB-mediated activities or shear banding would deteriorate mechanical properties of NC materials [2,11,12,13].

However, recent work revealed that composite structures with both NC and amorphous could exhibit superior mechanical properties. The compression stress of supra-nanometer-sized dual-phase glass-crystal Mg alloy could reach as high as 6.5 GPa [14]. Nanolaminates of crystalline Cu and amorphous Cu_3_Zr had an exceptionally high strength of ~1 GPa [15]. Additionally, embedded nanocrystals could improve the strength of nanoglass [16]. Further, amorphous intergranular films significantly promoted the structural stability of ng Ni-W alloy at elevated temperatures [17]. Most interestingly, as a special form of complex of nanostructure and metallic glass, nanograined metallic glass (NGMG) exhibited unusual mechanical properties [6]. The promotion of mechanical properties of NC or amorphous metallic material with a composite NC/amorphous structure mostly comes from suppression of shear localization upon external mechanical stimuli by the inclusion of NC structure into amorphous or amorphous into NC materials.

Ni-Mo coatings are of promising potential application, not only for their good electrocatalytic property for hydrogen evolution [18], but also characterized by their high corrosion resistance [19]. In addition, electrodeposited Ni-Mo alloys are also believed to be a promising alternative to hard chromium coatings for wear resistance without hazardous consequences [20]. According to Archard’s law, wear loss of materials is inversely proportional to their hardness [21], strengthening or hardening is highly desired for Ni-Mo coatings. One of the approaches to harden or strengthen metallic materials is to refine grain size following a traditional Hall–Petch relationship [22,23]; however, in our previous work in electrodeposited NC Ni-Mo alloys with different grain size, we found softening occurred when grain size was reduced below 10 nm, which was attributed to strain localization due to grain coarsening by GB activities [2]. Nevertheless, further investigation demonstrated that microhardness of NC Ni-Mo alloys was greatly promoted when GB activities was suppressed by either enhancing GB stability or lowering temperature [2,10]. Similarly, if GB activities or strain localization can be suppressed through introducing amorphous to NC Ni-Mo, mechanical performance of NC Ni-Mo would possibly be improved.

In our recent work in the synthesis of a series NG Ni-Mo alloys with different grain sizes by using direct current electrodeposition, it is found that grain size can be refined by tuning the atomic percentage of Mo in the alloy [2,24,25]. The minimum grain size achieved was 3.4 nm when Mo content was increased to 21.5 at. %. If the Mo content is increased higher than the value, amorphous phase could possibly be included to the NC structure before any second phase appears [26,27,28]. However, by using direct current deposition, the obtained Ni-Mo coating exhibited a poor surface appearance to provide any functional performance with further increasing Mo content. In the current work, pulsed electrodeposition will be employed to synthesize Ni-Mo deposit with high Mo content [27,29] with subsequent microstructure and chemistry characterization. Mechanical property will be evaluated by microhardness test. The underlying deformation mechanism will be discussed in terms of microstructure evolution and chemistry redistribution.

## 2. Materials and Methods

The electrolyte employed for pulse electrodeposition was composed of 60 g/L NiSO_4_·2H_2_O, 80 g/L Na_3_C_6_H_5_O_7_·2H_2_O and 3 g/L NaMoO_4_·2H_2_O, which was maintained at 35 °C with a pH value of 7.7 adjusted by ammonia. A mechanically polished copper disc with a diameter of 11 mm and a 99.99% pure platinum plate were employed as cathode and anode, respectively. A peak current density of 50 mA/cm^2^, with a pulse duration of 0.6 s and a duty cycle of 0.5 was introduced by using PGSTAT204M Autolab electrochemical workstation (METROHM, Herisau, Switzerland). Films with an average thickness of 70 μm were obtained after 5 h deposition.

Chemical composition of the deposit was determined by using the energy dispersive spectroscopy (EDS) technique on a Nova NanoSEM 430 (FEI Inc, Hillsboro, OR, USA) operated at 15 kV. At least 3 samples were examined. Structural characterization was conducted by using X-ray diffraction (XRD) performed on a Rigaku D/Max 2400 (Tokyo, Japan) operated at 100 mA and 40 kV with a Cu Kα source scanning at a step of 0.06°/s. In-plane transmission electron microscope (TEM) samples were prepared by mechanical grinding followed by electrochemical thinning at 20 V in a solution of perchloric acid and alcohol with a ratio of 1:9 at a temperature of −20 °C. Side-view TEM observation samples were prepared by focused ion beam (FIB) equipped on an FEI Verios 460 scanning electron microscopy (SEM, Hillsbora, OR, USA). Grain sizes were evaluated by using dark field TEM images through measuring intercept size of more than 300 grains and transforming into grain size by area fraction. TEM observation, high angle annular dark field-scanning transmission electron microscopy (HAADF-STEM), EDS mapping for compositional fluctuation were all acquired by using FEI Talos F200X (Hillsboro, OR, USA) operated at 200 kV.

Microhardness was evaluated on a Qness Q10A+ Micro-Hardness Tester (QATM, Golling, Austria) with a load of 50 g and a holding time of 10 s by at least 15 indentations.

## 3. Results and Discussion

### 3.1. Microstructure and Chemistry of the As-Deposited Ni-Mo Alloy

**Figure 1:**
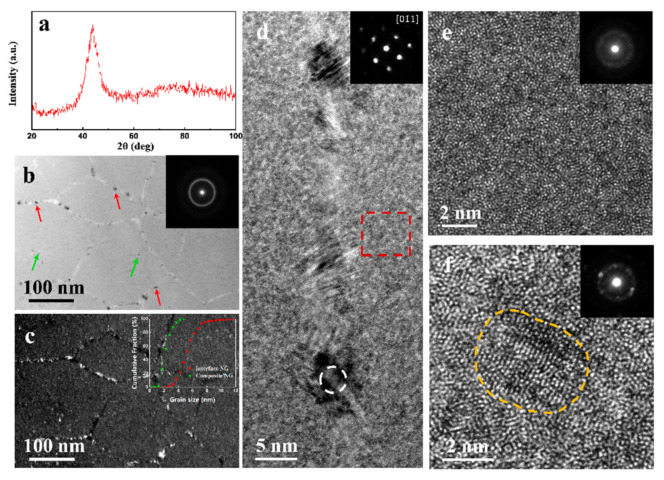
Microstructure of the as-deposited Ni-26Mo alloy. X-ray diffraction pattern (**a**) of the as-deposited Ni-26Mo. Plane view bright field (BF) (**b**) and dark filed (DF) (**c**) TEM images of the as-deposited Ni-26Mo. Insets in (**b**,**c**) are the corresponding selected area electron diffraction (SAED) and cumulative fraction of grain size of nanograins at nanocrystalline (NC) interfaces and mixed region of NC and amorphous as indicated by red and green arrows in (**b**), respectively. (**d**) A typical high-resolution TEM (HRTEM) image of NC interface with the inset being the nanobeam electron diffraction (NBED) pattern of a nanograin as designated by the white circle. Enlarged HRTEM images of amorphous (**e**) as outlined by red square in (**d**) and NC (**f**) in mixed region of NC and amorphous with the inset being their corresponding NBED patterns.

There is one broadened diffraction peak in the XRD pattern of the as-deposited Ni-Mo sample (Figure 1a), corresponding to a 2θ of 43.9°, lower than that of (111) peak of face centered cubic (fcc) pure Ni (44.51°), resulting from large Mo atoms inclusion to the Ni lattice.

Bright field (BF) TEM observations showed that the deposit was composed of polygonal crystalline-like regions divided by interfaces (Figure 1b). Those regions scales from about one to two hundred of nanometers, where very tiny scattered grains were discerned (green arrows). Continuous small grains were also spotted at interfaces (red arrows). Grains at or outside interfaces were observed more clearly in the dark filed (DF) TEM image (Figure 1c). The cumulative area fraction of grains at interfaces approached to larger grain sizes than those at divided regions (Inset of Figure 1c). A clear and a very diffusive halo ring were observed in the inset selected area electron diffraction (SAED) pattern in Figure 1b. Interfaces were composed of a succession of tiny grains that had an average gain size of 4.9 nm under high-resolution TEM (HRTEM) (Figure 1d). Single crystal diffraction pattern with an fcc structure was obtained by using nanobeam electron diffraction (NBED), which was taken at the interface outlined by the white circle (Inset in Figure 1d). By contrast, regions divided by interfaces were composed of a glass-like structure with scattered extremely fine nanograins, as presented in Figure 1d–f. There was no discernable lattice fringe under HRTEM of the red square in Figure 1d verified by the inset NBED, exhibiting a glass-like structure (Figure 1e). However, NBEDs from some interface divided regions exhibited both glass halo and diffraction spots (Figure 1f), presenting a mixed structure of NC and amorphous. The average grain size of nanograins at mixed regions was 2.5 nm, smaller than that at interfaces (4.9 nm).

**Figure 2:**
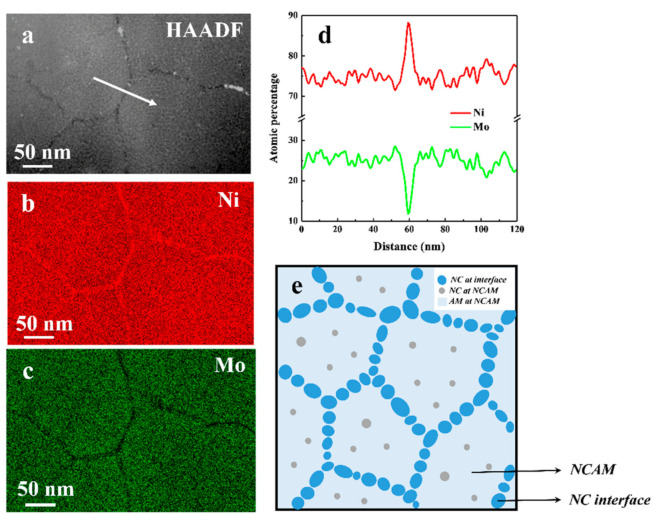
Chemistry analysis of the as-deposited Ni-26Mo alloy. (**a**) A high angle annular dark field (HAADF) image of Ni-26Mo. Energy dispersive spectroscopy (EDS) mapping of nickel (**b**) and molybdenum (**c**) in the same region of (**a**), indicating an obvious enrichment of Ni and depletion of Mo at NC interface. (**d**) Composition profile of Ni and Mo across NC interface as indicated by the white arrow in (**a**), showing the atomic percentage of Ni is 88 with that of Mo is 12. (**e**) A schematic illustration of nanocrystalline/nanocrystalline and amorphous (NC/NCAM) composite structure of Ni-26Mo.

The above analysis demonstrates that the obtained Ni-Mo alloy had a composite structure of NC and amorphous. Although EDS detection revealed the bulk Mo content in the Ni-Mo alloy is 26 ± 0.5 (Ni-26Mo), STEM-HAADF micrograph demonstrated a heterogeneous chemistry distribution (Figure 2a). EDS mapping of Ni and Mo of the same region as shown in Figure 2a, confirmed that there is an enrichment in nickel (Figure 2b) and a depletion of molybdenum (Figure 2c) at interfaces. One dimensional element distribution profiles of Ni and Mo from line scan across an interface indicated in Figure 2a revealed the concentration of Mo at NC/amorphous mixed regions fluctuated between 24 to 28 at. %, while that at the NC interface went down sharply to about 12 at. %, much lower than the bulk content (Figure 2d). Previous investigations demonstrated that the microstructure of electrodeposited Ni-based binary alloys could be tuned by controlling the content of alloy element [24,25]. The heterogeneity in alloy chemistry is closely related to the inhomogeneous microstructure.

A schematic illustration of the composite structure of Ni-26Mo is presented in Figure 2e, where NC interfaces composed of extremely fine nanograins (~4.9 nm) divide the microstructure into sub-micro-sized regions, which consist of amorphous (light-blue) and randomly-distributed tiny grains (gray dots)(~2.5 nm). A similar microstructure was observed in electrodeposited Ni-21.5 at. %W, which was nominally amorphous, but scattered with 5 and 8 nm fcc crystalline grains [30]. It is proposed that this microstructure be closely related to chemical fluctuation that might have come from mass consumption and transfer in the nucleation and growth in pulse electrocrystallization process. The structure of Ni-26Mo is similar to that of nanoglass, where interface and those in-between glassy particles have different chemical compositions [6]. However, Ni-26Mo is not fully amorphous, both interface and the glassy regions contain large amount of nanograins. The diffraction pattern shown in Figure 1a also reflects a superposition of the two parts of NC interfaces with a low Mo content and NC/amorphous mixed regions with high Mo content. The strong (111) diffraction pattern should partly come from low Mo content NC interfaces. In consideration of previous work on synthesizing extremely fine NC Ni-Mo samples by increasing Mo content [2,24,25], this composite structured Ni-26Mo demonstrated a crossover from NC to amorphous when tuning up Mo atomic percentage. For simplicity, the structure of Ni-26Mo was named NC/NCAM to stand for interface of nanocrystalline (NC) and mixed region of nanocrystalline and amorphous (NCAM), respectively.

### 3.2. Microhardness

**Figure 3:**
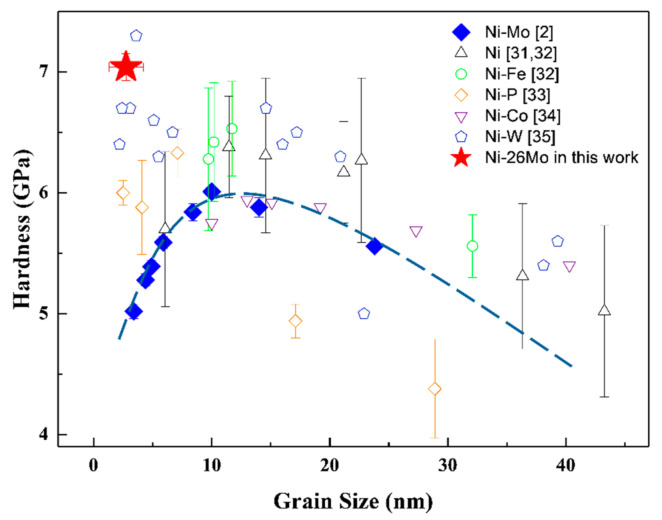
Hardness in NC/NCAM Ni-Mo alloy. Variation of microhardness data with grain size of electrodeposited NC Ni and Ni alloys from literature. The red star represents the microhardness of Ni-26Mo. The blue dotted line representing a deviation from Hall-Petch when grain size goes below about 10 nm.

Figure 3 presents hardness values of electrodeposited NC pure Ni and some nickel alloys [2,31,32,33,34,35]. Despite the experimental difference in hardness measurements, generally the microhardness variation against grain size followed the classical Hall–Petch before hardness decreased when grain size was reduced below 10 nm. For Ni-Mo alloy [2], specifically, the microhardness increased to a maximum value of 6.01 ± 0.04 GPa at 10 nm and decreased gradually to 5.02 ± 0.06 GPa when grain size fell down to 3.4 nm (Ni-21.5Mo). The microhardness of this NC/NCAM Ni-Mo was 7.04 ± 0.11 GPa, as the red star in the figure represents, being 40% higher than Ni-21.5Mo. Except one point from Ni-W [35], the value was also higher than other NC Ni and nickel alloys at the “strongest size” [32,33,34,36].

In homogeneous structured extremely fine NC alloys, plastic deformation was mainly accommodated by GB-mediated activities when significant grain coarsening and softening were generally observed [2,5]. The evidently enhanced hardness infers that grain coarsening or softening might have been suppressed by the crossover structure from nanocrystalline to amorphous. To clarify the issue, microstructure and chemistry of the indented sample were characterized and analyzed.

### 3.3. Microstructure and Chemistry of the Indented Ni-26Mo

**Figure 4:**
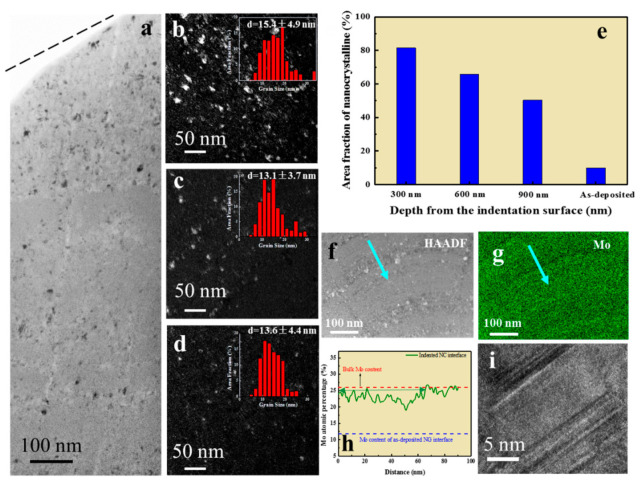
Microstructure and chemistry of the deformed Ni-26Mo. (**a**) A cross-section BF image of the Ni-26Mo after microhardness test by a load of 50 g. (**b**–**d**) are the corresponding DF images at a depth of 300, 600, and 900 nm from the indented surface of (**a**) with the inset being the statistical grain size distribution from corresponding DFs, black dotted line in (**a**) marks the indented surface. (**e**) Area fraction of NC at different depth from the indented surface after microhardness test. (**f**) An HAADF image taken from cross section of indented Ni-26Mo. (**g**) EDS mapping of solute atom Mo in the same area of (**f**), presenting redistribution of Mo. (**h**) Compositional profile of Mo underneath an indented surface across NC interface as indicated by the light blue arrow (Indented NC interface) in (**f**,**g**). The red and blue segment line in (**h**) represent the bulk and the initial NC interface Mo content, respectively. (**i**) An HRTEM image of a grain from an area right beneath the indented surface, presenting multiple parallel twins and stacking faults across the grain.

More nanograins emerged in the deformed microstructure, as shown in the cross-sectional BF TEM image after microhardness test (Figure 4a), and these newly-formed grains tended to form near the original NC interfaces. Grain sizes from DFs at different depth (Figure 4b–d) are 15.4 ± 4.9 nm, 13.1 ± 3.7 nm, and 13.6 ± 4.4 nm, respectively, illustrating slight grain coarsening under indentation. Additionally, no shear band was observed.

Area fraction of nanograins derived from DFs in Figure 4b–d in the deformed structure became progressively larger from bottom to top surface, as shown in Figure 4e. At a depth of 900 nm, nanograins accounts for 50% of area while the value goes up to 80% at a depth of 300 nm, indicating 70% of the nanograins was derived from indentation in comparison with the 10% in the as-deposited sample. Hardness indenter confined plasticity to a small volume of material under the tip, which was subjected to severe plastic deformation. Significantly progressively increased area fraction of nanograins with depth inferred that plastic deformation induced evident crystallization in NC/NCAM structured Ni-26Mo.

HAADF-STEM images shown in Figure 4f confirmed crystallization occurred in the vicinity of original NC interface. EDS mapping of the solute atoms indicated a heterogeneous Mo distribution (Figure 4g) with Mo depletion in NC region. However, elemental distribution profile showed that the width of the NC interface was about 70 nm, much wider than that of the as-deposited (4.9 nm). Although Mo atomic percentage in the indented NC interface was still lower than that of the bulk Ni-26Mo, the lowest Mo content in the profile was 17–18 at. %, which was 5–6 at. % higher than that of as-deposited NC interfaces (Figure 4h). Clearly, crystallization was accompanied by Mo diffusion from mixed regions to interfaces.

In addition, stripped contrast of twins and stacking faults appeared in some grains right beneath the indented surface, verified by the HRTEM image (Figure 4i), where multiple parallel stacking faults and twin were clearly observed. A similar characterized feature was observed in extremely fine NC Ni-Mo alloy when GB activities was suppressed [2], which was believed to be resulted from nucleation of partial dislocations from GBs.

In Ni-26Mo alloy with a composite structure of NC/NCAM, plastic deformation should have started from NC interfaces, which was probably softer than the surrounding material due to a lower Mo content, quite similar to nanoglass, where shear bands usually nucleate from soft interfacial regions [37]. This was evidenced by the fact that crystallization in Ni-26Mo was usually observed in the vicinity of NC interfaces, especially at the region away from the top surface where plastic strain is small (Figure 4a). However, as nanograins at interfaces were surrounded by amorphous phase, GB activities of these extremely fine nanograins should have been suppressed because the surrounding amorphous phase has to be crystallized first before any GB activities could proceed. This was supported by the increasingly enlarged area fraction of nanograins with decreasing depth from the indented top surface (Figure 4e). Also, the average grain size near the top surface was around 15 nm, much less than the 150 nm observed in a NC Ni-Mo alloy with an initial grain size of 4.9 nm [2]. Further, suppression of GB activities was evidenced by the appearance of multiple stacking faults and twins in some grains close to the indented surface, left behind by nucleation and propagation of partial dislocations from GBs [2,38].

On the other hand, different from fully amorphous alloys, where plastic deformation induced crystallization was generally found at or around shear bands [39,40,41], plastic deformation in Ni-26Mo was mainly accommodated by crystallization rather than shear banding. It was well documented that crystallization could induce deformation in metallic glasses [39,40,41], as well as disperse strain localization [42]. Besides, chemistry analysis revealed that plastic deformation induced crystallization in Ni-26Mo was accompanied by Mo diffusion from NCAM mixed region to NC interfaces (Figure 4f–h), which might have also reduced the tendency of strain localization. Therefore, no shear bands were observed in the indented deformed microstructure of the Ni-26Mo alloy.

The present work demonstrates an example of a non-equilibrium alloy with a microstructure crossover from NC to amorphous which exhibits a superior hardness to its NC counterparts due to the suppression of GB-mediated activities and shear banding that would otherwise degrade mechanical performances. Analogous to nanoglass, mechanical properties of NC alloys can be enhanced through compositional tuning by introducing a heterogeneous architecture of NC and amorphous.

## 4. Conclusions

1. By employing pulse electrodeposition, a Ni-26 Mo alloy was synthesized. Microstructural characterization revealed that the alloy was composed of mixed regions of amorphous and nanograins divided by NC interface network.
2. Chemical analysis uncovered that the composite structure correlated to a chemical fluctuation betweenmixed regions corresponding to a higher Mo content and the interfaces to a lower Mo atomic percentage.
3. Microhardness of the composite structured Ni-Mo was 7.04 ± 0.11 GPa, which was up to 40% higher than its extremely fine NC counterparts.
4. Microstructure characterization and chemical analysis on the indented sample indicated that plastic deformation of the composite was mainly accommodated by crystallization. This crystallization process was accompanied by atomic diffusion, which not only suppressed GB-mediated activities but also reduced the tendency of strain localization by shear banding.

## Data Availability

Data available on request due to restrictions eg privacy or ethical.
